# Artificial Neural Networks for IoT-Enabled Smart Applications: Recent Trends

**DOI:** 10.3390/s23104853

**Published:** 2023-05-18

**Authors:** Andrei Velichko, Dmitry Korzun, Alexander Meigal

**Affiliations:** 1 Institute of Physics and Technology, Petrozavodsk State University, 33 Lenin Ave., 185910 Petrozavodsk, Russia; 2 Department of Computer Science, Institute of Mathematics and Information Technology, Petrozavodsk State University, 33 Lenin Ave., 185910 Petrozavodsk, Russia; dkorzun@cs.karelia.ru; 3 Medical Institute, Petrozavodsk State University, 33 Lenin Ave., 185910 Petrozavodsk, Russia; meigal@petrsu.ru

In the age of neural networks and the Internet of Things (IoT), the search for new neural network architectures capable of operating on devices with limited computing power and small memory size is becoming an urgent agenda. Trends in the development of artificial intelligence (AI) applications in the field of the Internet of Things include smart healthcare services [1,2,3,4,5], smart object-recognition [6,7,8], smart environment monitoring [9,10], and smart disaster rescue [11]. Traditionally, such applications operate in real time. For example, security camera-based object-recognition tasks operate with detection intervals of 500 ms to capture and respond to target events. The data processing of human health and physiological parameters from different sensors (heart rate monitoring, glucose monitoring, oxygen saturation, etc.) generally requires immediate processing. Often, commercial smart IoT devices transfer information to the cloud for subsequent intelligent processing. However, stable network connections are not available everywhere, and it is a limitation for meeting real-time requirements. The solution to this problem can be the execution of information processing using neural networks installed directly on IoT devices. In this case, the quality of the Internet connection would not have a significant impact. Enabling artificial intelligence directly on the device is a challenge because of the limited computing power and small memory size of IoT devices. Frequently, smart applications need to run on a lightweight OS with a minimal set of libraries that imposes limitations on the operation of resource-intensive neural networks.

AI technologies for IoT devices and edge computing are demanded in mobile healthcare (m-Health), as well as in close application domains. Ambient intelligence (AmI) environments are constructed in IoT domains to provide smart services based on real-time analysis of human cognitive and motion functions. This Special Issue focuses on recent developments in the constantly growing application field of computing technologies and artificial intelligence algorithms. It includes new approaches to the organization of artificial intelligence on edge devices, as well as the organization of modular, feed forward, distributed, reservoir, recurrent, convolutional, and deep neural networks for various IoT-enabled smart applications. The guest editors are Andrei Velichko (Institute of Physics and Technology), Dmitry Korzun (Institute of Mathematics and Information Technology), and и Alexander Meigal (Medical Institute); they are all from Petrozavodsk State University, Russia.

The Special Issue collects eleven papers to provide a multi-domain overview of the trends and developments in the edge computing and starts with the illustration of achievements in smart healthcare services. The digitalization of healthcare driven by the IoT and AmI leads to the effective use of sensors, when various parameters of the human body are instantly tracked and processed in daily life [1,2]. The concept of machine learning sensors is applied to the diagnosis of COVID-19 as IoT application in healthcare and ambient assisted living. An important task is to determine the status of infection with COVID-19 using various diagnostic tests. This study provides a fast, reliable, and cost-effective alternative tool for diagnosing COVID-19 based on routine blood values measured at clinic admission. Popular machine learning classifiers were studied and their important features were identified to ensure the high accuracy of disease diagnostics. The study [2] continues the topic of COVID-19 diagnostic and reviews the routine blood values using a backward feature elimination algorithm and the LogNNet reservoir neural network. The proposed method reduces the negative pressures on the health sector and helps doctors to understand the pathogenesis of COVID-19 using the key blood values. The method demonstrates high opportunity of the LogNNet network to be applied in IoT smart applications.

An IoT-enabled system to monitor gait in subjects with Parkinson’s disease is presented in study [3]. Parkinson’s disease is one of the most studied pathologies in the field of neurology and is suitable for application of science-intensive study methods, virtual reality technologies and even robotics. Wearable sensors and IoT-enabled technologies look promising for monitoring motor activity and gait in Parkinson’s disease patients. The gait was measured and characterized with help of the accelerometer signal acquired from inertial measurement unit of a smartphone attached to the head during the timed up and go test. Smartphones as a measuring device are well suited for the creation of IoT-enabled systems. The use of accelerometer signals received from a smartphone inertial measurement unit creates high potential for AI-supported systems and makes the proposed method applicable not only in healthcare laboratories but in the daily life settings.

Smart healthcare applications, the Internet of Things (IoT), and artificial intelligence are arguably the most appropriate customized solutions for such shortcomings of traditional healthcare systems, such as long waiting times, unnecessary long trips to health centers, high costs, and mandatory periodic doctor visits. The comprehensive literature review [4] determines the impact of IoT, AI, various communication technologies, sensor networks, and disease detection in Cardiac healthcare. The results of the review show that deep learning is emerging as a promising technology along with the combination of IoT in the field of cardiac care with increased accuracy and real-time clinical monitoring. In addition, this study points out the main advantages and major challenges of e-cardiology in the areas of IoT and AI.

Another illustration of the effective application of neural networks in healthcare is presented in study [5]. Speech is a complex mechanism that allows us to communicate our needs, desires, and thoughts. In some cases of nervous dysfunction, this ability is severely affected, making daily activities that require communication difficult. This study explores various options for an intelligent imaginary speech recognition system that can be installed on low-cost devices with limited resources. The authors used a method based on covariance in the frequency domain, which performed better than other methods in the time domain. Several architectures of convolutional neural networks have been studied and it has been demonstrated that a more complex architecture does not necessarily lead to better results. The results prove that cheap IoT devices can be effectively used in speech recognition and contribute to the development of IoT-enabled smart applications.

The realm of smart object-recognition applications of AI systems is presented in the subsequent articles [6,7,8]. Driver assistants have become a more and more popular class of smart IoT-enabled smart applications, as illustrated in study [6] that detects distracting actions in driver activities. According to the World Health Organization, the increase in car accidents is a major problem in today’s transportation systems, and is the eighth leading cause of death worldwide. More than 80% of traffic accidents are caused by distraction while driving. A practical approach to solving this problem is to introduce quantitative indicators of driver activity and develop a classification system that identifies distracting activities. Authors implemented a portfolio of different ensemble deep learning models that have been proven to effectively classify driver distractions and provide in-vehicle recommendations to minimize distraction levels and improve safety. Another lifesaving application based on deep learning is a child drowning prevention system [7]. The proposed deep convolutional neural networks-based models can be used to automatically detect the possible distractions of a caregiver who is supervising a child and generate alerts to warn them. The system was tested in a swimming pool, and we think it could be implemented in natural water reservoirs to avoid possible child drowning. Such smart applications for the rapid detection of dangerous situations are of critical importance, as they are able to observe persons and their activity more effectively than humans.

For the IoT-enabled smart applications, point clouds are one of the most widely used data formats created by depth sensors. Research on feature extraction from disordered and irregular point cloud data has advanced recently. The overview [8] of the different types of models is presented, and studies of point clouds and remote sensing problems have been carried out using deep learning methods. It is concluded that convolutional neural networks achieve the best performance in various remote sensing applications that operate directly with raw cloud data. The lightweight models are especially important for IoT edge computing.

The research direction of smart environment monitoring is presented by the study [9], in which a model of artificial neural network was integrated into a Raspberry Pi-based sensor to implement edge computing for hourly river level prediction. The model that consists of a three-layer perceptron is able to predict river levels with a high degree of accuracy using only previously observed water levels, precipitation, and runoff information as input, without the need for other hydrological and meteorological parameters. This study is a first attempt to combine real-time customized sensors and artificial neural network algorithms in practice. The model was built into a low-cost, open-source, and low-energy-consumption custom sensor to forecast the water level. A high level of model performance applied to real events, and the low-cost system is of interest for environmental monitoring. Another potential reference case for the development of smart IoT-enabled systems for environmental monitoring is presented in the study of determining the carcinogenicity of thousands of wide-variety classes of real-life exposure chemicals [10]. Authors have developed carcinogen prediction models based on the hybrid neural network deep learning method. The proposed model has a high potential for use in various IoT environmental projects.

Smart disaster rescue is presented by an interesting development of a wearable device for search dogs that recognizes the behavior of a dog when a victim is found, using deep learning models [11]. With their exceptional sense of smell and hearing, search and rescue dogs are important in first aid because they are able to locate a victim in conditions that are difficult for humans to reach. The authors propose an implementation of a wearable device that supports deep learning, including a base station, a mobile application, and a cloud infrastructure. The device can, firstly, track the activity, sounds and location of the search and rescue dog in real time, and, secondly, recognize and alert the rescue team whenever the dog spots a victim. For activity recognition, deep convolutional neural networks were used for classifying dog sounds, as well as inertial sensors. The developed deep learning models operated on a wearable IoT device. The functioning of the system was tested in two separate search and rescue scenarios, which allowed to successfully locate the victim and inform the rescue team in real time based on IoT technology.

In conclusion, this Special Issue illustrates advanced cases of using the AI technology for IoT-enabled smart applications. Each case demonstrates a promising trend for applying AI in IoT environments, making a step towards the effective use of modern technologies in our everyday life.
